# Headspace versus direct injection for the quantitation of methanol, ethyl acetate, and fusel oils in distilled spirits by gas chromatography – Flame ionization detection

**DOI:** 10.1016/j.mex.2023.102387

**Published:** 2023-09-21

**Authors:** Janel E. Owens, Luis E. Lowe

**Affiliations:** Department of Chemistry and Biochemistry, University of Colorado Springs, 1420 Austin Bluffs Parkway, Colorado Springs, CO 80918, United States

**Keywords:** Methanol, Ethyl acetate, Fusel oils, GC/FID, Distilled spirits, Headspace, No Sample Prep Headspace Analysis of Methanol, Ethanol, and Fusel Oils with Separation and Detection by GC/FID

## Abstract

Distilled spirits can be very complex in their sensory or organoleptic compounds. Of significant interest is determination of the concentration of methanol, ethyl acetate, and fusel oils, which include n-propanol, isobutanol, n-butanol, active amyl (2-methyl-1-butanol) and isoamyl (3-methyl-1-butanol) alcohols. Here, we describe a validated method for the analysis of these analytes using a headspace (HS) sampling unit coupled with a gas chromatograph fitted with a flame ionization detector (GC/FID) for profiling these analytes in distilled spirits (*n* = 26) obtained from local retailers. HS results were compared to the direct injection (DI) GC/FID protocol made available by the US Alcohol and Tobacco Tax and Trade Bureau (TTB), method SSD:TM:200 via correlation and Bland-Altman difference plots to demonstrate that HS-GC/FID is a valid alternative to the direct injection protocols described elsewhere.

• A method for the analysis of methanol, ethyl acetate, and fusel oils via headspace sampling coupled to a gas chromatograph fitted with a flame ionization detector (HS-GC/FID) is described.
• Samples required no pre-treatment beyond diluting 1 mL of distilled spirit in 4 mL water containing table salt, which resulted in a method with minimal inlet or column maintenance, little sample prepration, and a rapid run time with retention times under 7 min.
• Validation by comparing to established protocols using direct injection made available by the US Federal Tax and Trade Bureau (TTB).

## Graphical abstract

**Figure:**
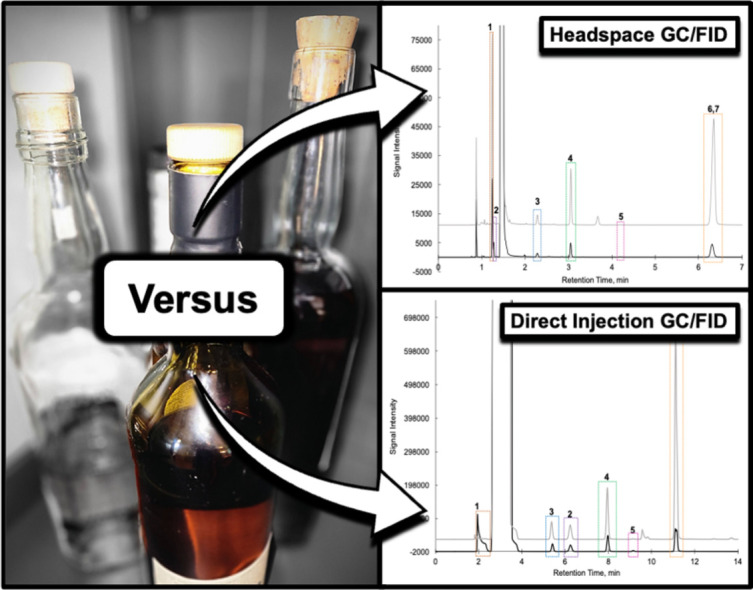

Specifications table

**Table:** 

Subject area:	Chemistry
More specific subject area:	Analytical Chemistry.
Name of your method:	No Sample Prep Headspace Analysis of Methanol, Ethanol, and Fusel Oils with Separation and Detection by GC/FID.
Name and reference of original method:	Y. Qin; J.A. Shin; K.T. Lee. Determination of acetaldehyde, methanol, and fusel oils in distilled liquors and sakes by headspace gas chromatography. *Food Sci. Biotechnol*. 2020, *29* (3): 331–337.
Resource availability:	Perkin-Elmer TurboMatrix HS40 headspace sampling unit Gas chromatograph with flame ionization detector Analytical column for separations

## Method details

Methanol, ethyl acetate, and fusel oils, which include n-propanol, isobutanol, n-butanol, active amyl (2-methyl-1-butanol) and isoamyl (3-methyl-1-butanol), are produced during fermentation and contribute to the sensory profile of distilled spirits [1]. Given the importance of these compounds to the flavor complexity, they are important to accurately quantitate to ensure quality and consistency in beverage products.

Previous groups have explored the use of solid-phase microextraction (SPME) coupled with gas chromatography-mass spectrometry (GC/MS) [2], headspace (HS) analysis with gas chromatography-flame ionization detection (GC/FID) [3,4], and direct injection (DI) GC/FID [1]. As described previously, there are issues associated with the direct injection of distilled spirits onto a capillary GC column including the need for more frequent maintenance and presence of interfering peaks [3,4]. Other manuscripts have described only the HS GC/FID method [2,3] without comparison to the DI GC/FID method [2,4] or with a limited pool of analyzed samples [2,3]. The method described here is useful for the separation and quantitation of methanol, ethyl acetate, and fusel oils without any sample pre-treatment by HS-GC/FID other that sample dilution into water containing 10% (w/v) sodium chloride. The developed HS-GC/FID method was assessed in terms of method parameters, accuracy, precision, and analyte concentration agreement with DI-GC/FID analyses for *n* = 26 distilled spirit samples.

## Materials and methods

### Chemicals and reagents

Omni-Pur ethanol (200 proof), active amyl alcohol (reagent grade), and isoamyl alcohol (reagent grade) were from Sigma-Aldrich (St. Louis, MO). Methanol (Optima-LC/MS grade), ethyl acetate (ACS grade), n-butanol (ACS grade), and n-propanol (ACS grade) were from Fisher Scientific (Fairlawn, NJ). Sodium chloride (ACS grade) and isobutanol (ACS grade) were from Mallinckrodt Baker (Phillipsburg, NJ). HPLC-grade 18 MΩ DDI water was produced via a Barnstead e-Pure system (Thermo Fisher Scientific). All other chemicals were from Fisher Scientific unless otherwise stated. The Omni-Pur ethanol was analyzed via the headspace GC/FID method before the preparation of the stock solution containing analytical standards to ensure purity, especially and to confirm low ethyl acetate content. Distilled spirits were obtained from local retailers.

### Preparation of standards

A stock solution of methanol, ethyl acetate, and fusel oils containing the following analytes (Table 1) was prepared in Omni-Pur ethanol (100 mL volumetric flask).

**Table 1:** Concentration of analytes in stock solution.

Analyte	CAS #	Amount (g or mL)	Concentration in Ethanol (mg/mL)
Methanol	67–56–1	20.0 mL	20.0%
Ethyl Acetate	141–78–6	2.00 g	20.0
n-Propanol	71–23–8	1.00 g	10.0
Isobutanol	78–83–1	2.00 g	20.0
n-Butanol	71–36–3	0.100 g	1.00
Active amyl alcohol	137–32–6	2.00 g	20.0
Isoamyl alcohol	123–51–3	2.00 g	20.0

This stock solution was used to create analytical standards at six levels (Table 2). These calibration standards were prepared in 25.0 mL volumetric flasks in ethanol/water (40/60, v/v). Analytical standards were stored at 8 °C when not in use and allowed to warm up to room temperature (20 °C) prior to pipetting into the 20 mL glass vials required of the headspace sampling unit.

**Table 2:** Analytes of the fusel oil panel with analytical concentrations at six levels.

Analyte	Level 1	Level 2	Level 3	Level 4	Level 5	Level 6
Ethyl Acetate	25 mg/L	50 mg/L	100 mg/L	200 mg/L	500 mg/L	1600 mg/L
Methanol	0.025 % (v/v)	0.050 % (v/v)	0.100 % (v/v)	0.200 % (v/v)	0.500 % (v/v)	1.600 % (v/v)
n-Propanol	12.5 mg/L	25 mg/L	50 mg/L	100 mg/L	250 mg/L	800 mg/L
Isobutanol	25 mg/L	50 mg/L	100 mg/L	200 mg/L	500 mg/L	1600 mg/L
n-Butanol	1.25 mg/L	2.50 mg/L	5.0 mg/L	10 mg/L	25 mg/L	80 mg/L
Amyls (Active + Isoamyl)	50 mg/L	100 mg/L	200 mg/L	400 mg/L	1000 mg/L	3200 mg/L

### HS-GC/FID parameters

A Perkin-Elmer TurboMatrix HS40 was utilized for all analyses. The method had the following temperature parameters: the oven for incubating glass vials was held at 75 °C, the transfer line from the headspace unit to the GC column was maintained at 170 °C, and the needle was held at 80 °C. The carrier gas (nitrogen) was held at 25.0 psi for all sample events. Sample vials were shaken and heated for 15 min and pressurized for 1.0 min prior to injection. The headspace gas was withdrawn for 0.1 min while the injection window was held to 0.02 min. The entire GC cycle was held for 22.0 min. The TurboMatrix HS40 allows for options with regards to operating mode (option selected: constant) and inject mode (option selected: time). The option for high-pressure injection was not utilized. Vials were vented upon completion of the heating and injection cycles.

**Fig. 1:**
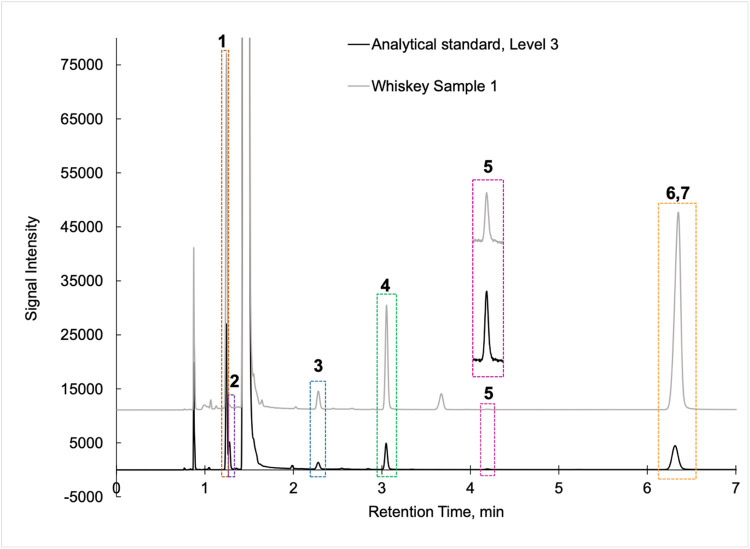
Example GC/FID chromatograms of analytical standard (black trace at Level 3, see Table 2) versus a whiskey sample (gray trace) by headspace sampling using the Perkin-Elmer TurboMatrix HS40 system with conditions described in the text. Analytes (1: Ethyl acetate; 2: Methanol; 3: n-Propanol; 4: Isobutanol; 5: n-Butanol; 6 and 7: Amyls – active and isoamyl) were reasonably well separated with the exceptions of analytes 1 and 2 and the co-elution of analytes 6 and 7.

Analytical standards and samples (Fig. 1) were injected onto a Restek Stabilwax-DA capillary column (30 m x 0.32 mm i.d., 0.25 µm film thickness) installed in gas chromatograph fitted with a flame ionization detector (GC/FID; Hewlett Packard 5890) operated with ChemStation software (*v* A.10.02). The injector was set to 180 °C to match the temperature setting of the Perkin-Elmer TurboMatrix HS40 transfer line. The GC oven was set to 45 °C (hold for 8.00 min) before ramping at 15.0 °C/min to 160 °C (hold for 0.50 min) for a total run time of 16.17 min. The column oven was equilibrated for 0.25 min between samples. Nitrogen was used as the carrier gas with a column head pressure of 25.0 psi. The FID was held at 275 °C.

### Preparation of calibration curves by HS-GC/FID

Analytical standards were prepared by pipetting 1.00 mL into a 20 mL glass vial. To this 1 mL volume, 4 mL of water containing sodium chloride (10 % w/v) were added via pipet. The glass vials were capped with a crimped septum-lined aluminum cap before being gently shaken by hand for 30 s. Vials were allowed to sit at room temperature on the autosampler tray until the standard was analyzed. Calibration curves for each analyte of Table 2 were prepared using the analyte area (*y*-axis) versus concentration. The curves were fit with 1/*x* weighting and prepared using the ChemStation software (Table 3). The limit of detection (LOD) and limit of quantitation (LOQ) were calculated as 3× the signal-to-noise (S/N) and 10× S*/*N. The n-butanol standard at Level 1 was 1.25 mg/L, which allowed for the determination of LOD and LOQ via S/N comparison. The LOD was 0.35 mg/L and LOQ was 1.17 mg/L. Given the much higher concentrations of the other fusel oil components in the analytical standards (Table 2) and their higher analytical sensitivity as measured by the slope (*m*) of the calibration lines (Table 3), these LOD and LOQ values were accepted for all other analytes analyzed via HS-GC/FID.

**Table 3:** Calibration curve data for analysis of fusel oil analytical standards by headspace (HS) GC/FID and direct injection (DI) GC/FID.

Analyte	Fitted Linear Regression for HS-GC/FID with (1/*x*) weighting	R^2^	Fitted Linear Regression for DI-GC/FID with (1/*x*) weighting	R^2^
Methanol	*y* = 65,850.8*x* + 280.2	0.9995	*y* = (9.965e+6)*x* + 10,461.5	0.9994
Ethyl acetate	*y* = 308.2*x* – 246.9	0.9997	*y* = 1523.0*x* + 1351.6	0.9996
n-Propanol	*y* = 51.3*x* + 240.2	0.9999	*y* = 2340.1*x* + 10,290.5	0.9990
Isobutanol	*y* = 101.2*x* + 1.7	0.9998	*y* = 2763.5*x*–1183.7	0.9993
n-Butanol	*y* = 70.7*x* – 3.6	0.9997	*y* = 2663.74*x*–553.3	0.9988
Amyls (Active + Isoamyl)	*y* = 112.0*x* + 63.4	0.9998	*y* = 2795.7*x*–3868.6	0.9993

### Analysis of samples by HS-GC/FID

To analyze distilled spirits (*n* = 26), including whiskey, bourbon, rum, tequila, cachaça, and gin, 1.00 mL of the sample was pipetted into a 20 mL glass vial. Four mL of water containing sodium chloride (10 % w/v) were added. The vials were capped, shaken by hand for 30 s, and allowed to sit at room temperature (20 °C) prior to analysis.

### Method validation – Spike and recovery by HS-GC/FID

To assess the accuracy and precision of the headspace method for quantitation of methanol, ethyl acetate, and fusel oil analytes, we spiked *n* = 3 samples (rum, gin, and cachaça) at two different spiking levels (Table 4). The concentrations of the spiked samples were compared against the concentrations of the fusel oil analytes in unspiked samples of these three distilled spirits and normalized by the spike amount (Eq. (1)) and the% relative standard deviation (% RSD) calculated for method precision. Samples were then analyzed on three different days over a period of ten days and the results pooled together to demonstrate accuracy and precision of the method.

(1) $$\%\;Recovery=100\;\times \;\left(\frac{Conc_{spiked\;sample}-\;Conc_{unspiked\;sample}}{Amount\;added}\right)$$

**Table 4:** Mean% Recovery (% RSD) of fusel oil spikes added to *n* = 3 different distilled spirits at two spiking levels.

Analyte	Spike Level A	Mean% Recovery (% RSD) for *n* = 7 replicates spiked at Level A	Spike Level B	Mean% Recovery (% RSD) for *n* = 3 replicates spiked at Level B
Methanol	0.050 %	92 (18.1)	0.025%	95 (12)
Ethyl acetate	50.0 mg/L	96.8 (11.5)	25 mg/L	124 (23)
n-Propanol	25.0 mg/L	104 (14.9)	12.5 mg/L	148 (15)
Isobutanol	50.0 mg/L	112 (11.7)	25 mg/L	119 (8.4)
n-Butanol	2.50 mg/L	106 (9.07)	1.25 mg/L	104 (13.4)
Amyls (Active + Isoamyl)	100 mg/L	114 (12.2)	50.0 mg/L	118 (23.7)

### Analysis of samples by DI-GC/FID

**Fig. 2:**
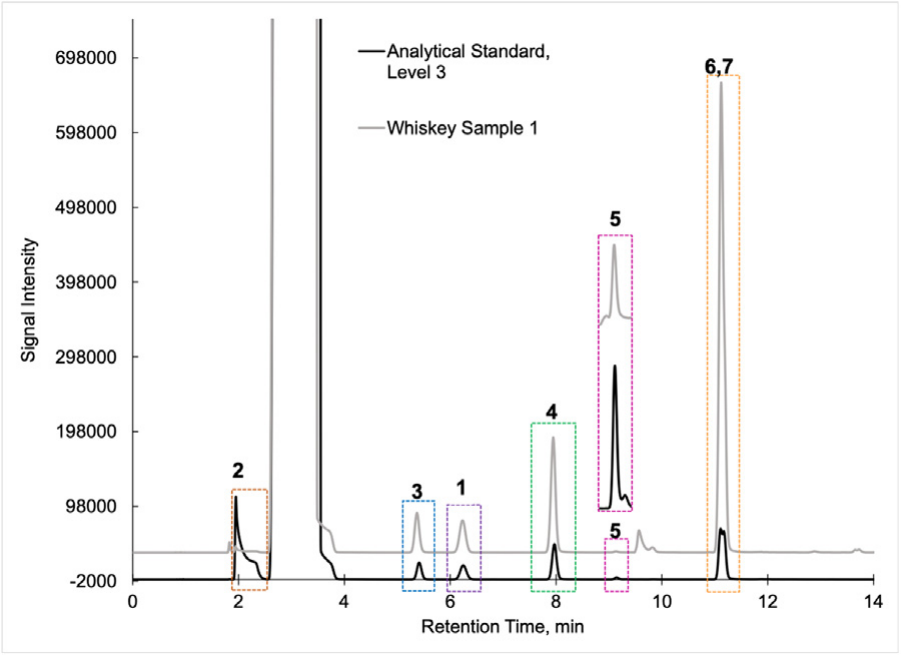
Example GC/FID chromatograms of analytical standard (black trace at Level 3, see Table 2) versus a whiskey sample (gray trace) by direct injection without sample preparation as described in the text. Note the different elution order than Fig. 1 where analytes (2: Methanol; 3: n-Propanol; 1: Ethyl acetate; 4: Isobutanol; 5: n-Butanol; 6 and 7: Amyls – active and isoamyl) were well separated.

To validate the determined concentrations of fusel oils in the distilled spirits (*n* = 26) included in this small study, the analytical standards of Table 2 and samples were analyzed on a different GC/FID system (Hewlett Packard 5890) fitted with an Agilent Technologies DB-624 megabore capillary column (30 m x 0.530 mm, 3.0 µm film thickness) with the following conditions using a where 1.0 μL of the analytical standard or sample was injected directly on column without any sample preparation (Fig. 2). The injector was set to 250 °C. The initial GC oven was set to 40 °C (hold for 6.00 min) before ramping at 10.0 °C/min to 220 °C (hold for 1.00 min) for a total run time of 27.0 min. The column was equilibrated for 0.10 min between samples. Nitrogen was used as the carrier gas with a column head pressure of 3.6 psi.

### Preparation of calibration curves by DI-GC/FID

Analytical standards (Table 2) were prepared by pipetting 1.00 mL into amber glass 2.0 mL autosampler vials with a PTFE-lined septum fitted into a screw-cap. Autosampler vials were allowed to sit at room temperature on the autosampler tray until the standard was analyzed. Calibration curves for each analyte of Table 2 were prepared using the analyte area (*y*-axis) versus concentration. The curves were fit with 1/*x* weighting and prepared using the ChemStation software *v* A.10.02 (Table 3). The LOD, LOQ, and calibration range for methanol, ethyl acetate, and fusel oil analytes were previously reported by the US Federal Alcohol and Tobacco Tax and Trade Bureau (TTB) method SSD: TM:200, rev 9 [1].

### Comparison of HS-GC/FID and DI-GC/FID

Quantitative results of the seven analytes as determined via the two methods (HS versus DI) were compared first by comparing correlation and range values. In terms of correlation and agreement between the two methods, the slope of the fitted regression line should be close to a value of 1 while the intercept should approximate 0. The *R^2^* value demonstrates the strength of the relation between the two datasets and not their agreement. For all analytes in this study that were compared via the two methods, strong linearity, a slope near unity, and intercept near 0 were observed (Table 5). However, these datasets were generally normally distributed by the d'Agostino-Pearson test (exceptions were for the analytes of methanol and isobutanol) [5]. As such, the datasets were compared via Bland-Altman comparison plots, which are useful for comparing the resultant data from two methods.

**Table 5:** Correlation comparison of quantitative results by two methods (headspace, HS) versus direct injection (DI) for the analyses of seven fusel oil analytes in distilled spirits (*n* = 26).

Analyte (*n*)	*R^2^*	Slope (*m*)	Intercept (*b*)	Range (HS)	Range (DI)
Methanol	0.8910	0.7553	0.0019	Not detected (ND) – 0.15 %	ND - 0.12 %
Ethyl Acetate	0.9768	0.8752	0.1416	2.1 mg/L – 546.1 mg/L	ND – 529.0 mg/L
n-Propanol	0.9271	1.0283	−10.7200	ND – 296.5 mg/L	ND – 338.1 mg/L
Isobutanol	0.9851	0.8679	−0.7119	ND – 1235.2 mg/L	ND – 1100.7 mg/L
n-Butanol	0.9776	0.9071	0.0058	ND – 15.2 mg/L	ND – 12.8 mg/L
Amyls (Active + Isoamyl)	0.9865	0.8813	−2.8472	ND – 2350.7 mg/L	ND – 2242.9 mg/L

**Fig. 3:**
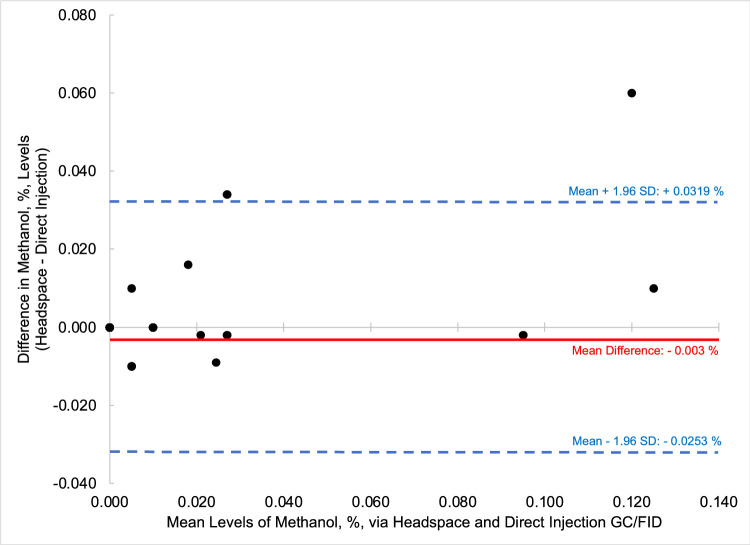
Bland-Altman difference plot for the comparison of methanol (measured as a v/v%) determined via HS-GC/FID – DI-GC/FID (*y*-axis) versus the mean methanol (%, v/v) levels determined by the two methods. The red line indicates the mean difference in results by the two methods, or −0.003%. To provide context for this mean difference, this is 12 % of the lowest analytical standard, 0.025 % (Table 2) or 16% of the mean methanol concentration for the pooled samples (HS + DI), which was 0.020%. Of the *n* = 26 distilled spirits samples analyzed, two samples had mean differences outside of the limits of agreement, mean difference ± 1.96 standard deviation (SD).

**Fig. 4:**
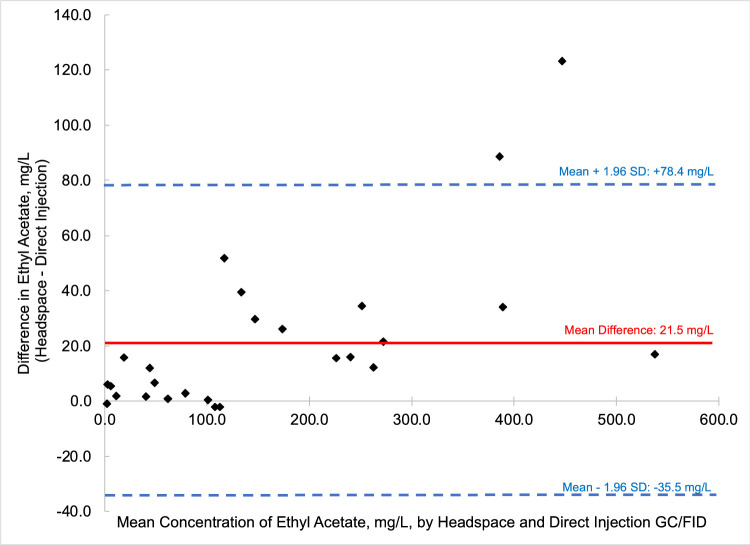
Bland-Altman difference plot for the comparison of ethyl acetate (measured as mg/L) determined via HS-GC/FID – DI-GC/FID (*y*-axis) versus the mean ethyl acetate (mg/L) levels determined by the two methods. The red line indicates the mean difference in results by the two methods, or 21.5 mg/L. To provide context for this mean difference, this is below the lowest analytical standard, 25 mg/L (Table 2) or 13 % of the mean for the pooled datasets (HS + DI), which was 162 mg/L. Of the *n* = 26 distilled spirits samples analyzed, two samples had mean differences outside of the limits of agreement, mean difference ± 1.96 standard deviation (SD).

**Fig. 5:**
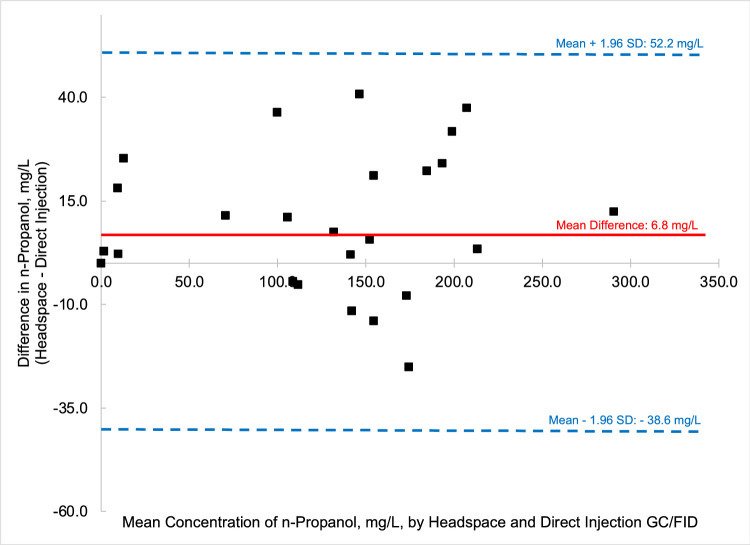
Bland-Altman difference plot for the comparison of n-propanol (measured as mg/L) determined via HS-GC/FID – DI-GC/FID (*y*-axis) versus the mean n-propanol (mg/L) levels determined by the two methods. The red line indicates the mean difference in results by the two methods, or 6.8 mg/L. To provide context for this mean difference, this is below the lowest analytical standard, 12.5 mg/L (Table 2), or 5.1% of the mean concentration for the pooled datasets (HS + DI), where the samples had an average concentration of 134 mg/L n-propanol. Of the *n* = 26 distilled spirits samples analyzed, no samples had mean differences outside of the limits of agreement, mean difference ± 1.96 standard deviation (SD).

**Fig. 6:**
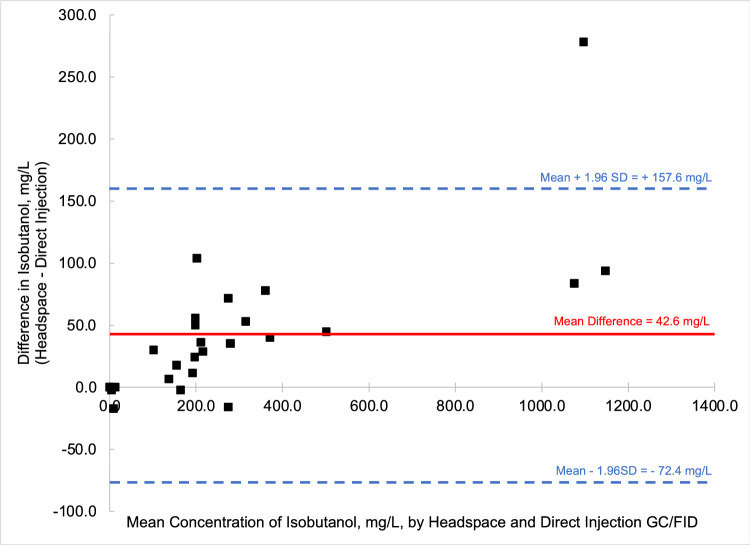
Bland-Altman difference plot for the comparison of isobutanol (measured as mg/L) determined via HS-GC/FID – DI-GC/FID (*y*-axis) versus the mean isobutanol (mg/L) levels determined by the two methods. The red line indicates the mean difference in results by the two methods, or 42.6 mg/L. The mean of the pooled datasets was 295.9 mg/L isobutanol, indicating that the mean difference between these two methods was 14 % relative to this pooled mean. Of the *n* = 26 distilled spirits samples analyzed, one sample had a mean difference outside of the limits of agreement, mean difference ± 1.96 standard deviation (SD).

**Fig. 7:**
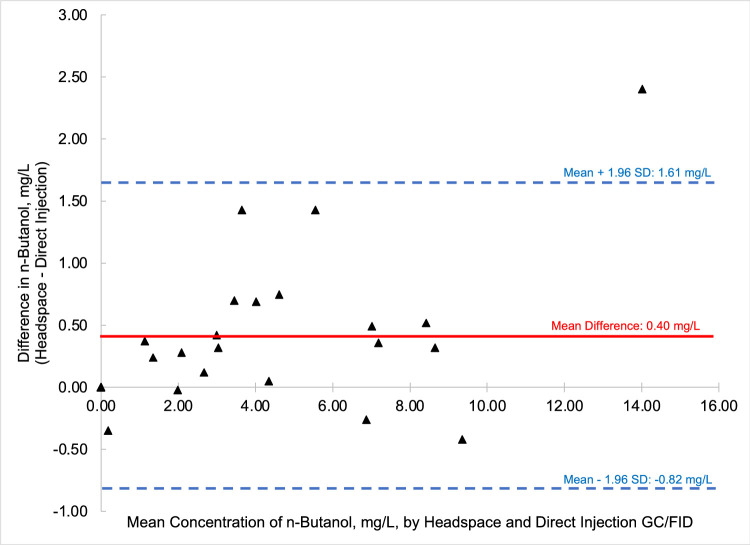
Bland-Altman difference plot for the comparison of n-butanol (measured as mg/L) determined via HS-GC/FID – DI-GC/FID (*y*-axis) versus the mean n-butanol (mg/L) levels determined by the two methods. The red line indicates the mean difference in results by the two methods, or 0.40 mg/L. The mean concentration of the pooled datasets (HS + DI) was 4.10 mg/L, and so the mean difference between the two method was 9.6% relative to the mean of the pooled samples. Of the *n* = 25 distilled spirits samples analyzed, one sample had a mean difference outside of the limits of agreement, mean difference ± 1.96 standard deviation (SD). One sample was deleted from the dataset as an outlier owing to an interferent at the n-butanol retention time, which resulted in a determined concentration of 233 mg/L, which far exceeded the high analytical standard.

**Fig. 8:**
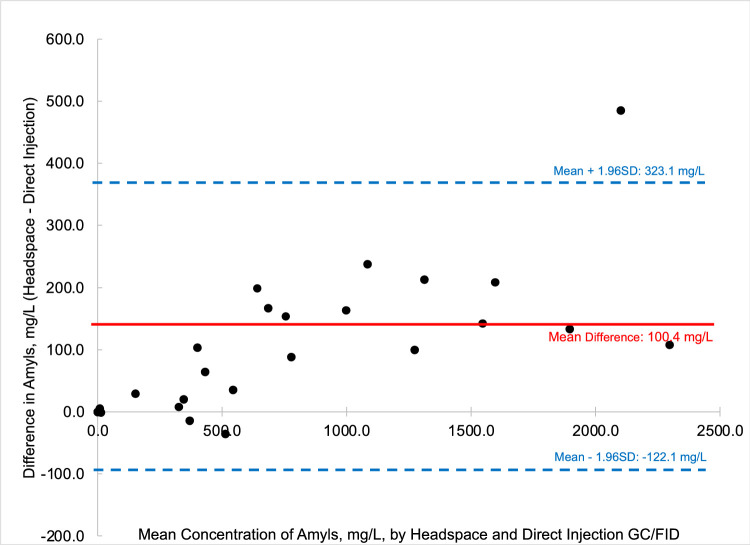
Bland-Altman difference plot for the comparison of amyls (measured as mg/L) determined via HS-GC/FID – DI-GC/FID (*y*-axis) versus the mean amyls (mg/L) levels determined by the two methods. The red line indicates the mean difference in results by the two methods, or 100.4 mg/L, which is 13 % relative to the mean concentration of the pooled datasets (HS + DI), which was 772.4 mg/L. Of the *n* = 26 distilled spirits samples analyzed, one sample had a mean difference outside of the limits of agreement, mean difference ± 1.96 standard deviation (SD).

To compare the quantified fusel oils in the *n* = 26 samples of distilled spirits by the two methods, that is HS-GC/FID versus DI-GC/FID, Bland-Altman comparison plots were generated for each analyte. Bland-Altman plots allow for the easy comparison between two methods by comparing the difference of the two methods (*y*-axis) versus the mean value of the two methods (*x*-axis). Most data points should then fall between ± 1.96 standard deviations of the mean difference to be comparable [6]. Fig. 3 through Fig. 4, Fig. 5, Fig. 6, Fig. 7, Fig. 8 demonstrate the excellent agreement between the two methods for the analysis of the methanol, ethyl acetate, and fusel oil analytes of the present study.

In summary, we present a validated headspace GC/FID method that allows for the facile determination of methanol, ethyl acetate, and fusel oils in distilled spirits. This work is important because it compares two different analytical methods (HS-GC/FID vs DI-GC/FID) in which different separation columns were utilized. We carried out spike and recovery studies at two different spiking levels with multiple replicates across three different spirit types to assess the accuracy and precision of the method, and then employed the methods for analysis of a large pool of distilled spirits. Results were accurate and precise for the HS-GC/FID method (see Table 3). Quantitative levels of the analytes in the distilled spirits by the two methods were compared via Bland-Altman difference plots (Fig. 3, Fig. 4, Fig. 5, Fig. 6, Fig. 7, Fig. 8) and demonstrated excellent comparability for all analytes with mean differences < 15% relative to the pooled datasets for a given analyte. While the two methods are comparable in cost for purchase of the analytical standards and chemical reagents, the methods diverge in terms of the maintenance required to generate and maintain high quality data. The headspace method offers a clear advantage for reduced inlet maintenance whereas the use of a glass liner in our direct injection GC/FID required that we be cognizant of number of injections on the system to ensure that maintenance was scheduled and completed. The direct injection system is preferred for its simple sample preparation: the samples are pipetted into amber glass autosampler vials and sealed to await injection. The headspace unit utilized 20 mL glass vials that could be reused, though the rubber septa and aluminum crimp-top lids were single-use only. Had we fitted the headspace system with a different column (note that we used a Restek Stabiliwax-DA, which has similar properties to an HP-FFAP column for separation of acids), we may have witnessed better separation between the methanol and ethyl acetate (Fig. 1), though the shape of the methanol peak on a DB-WAX column (Fig. 2) encouraged us to try other column stationary phases to investigate how that peak shape could be improved. Given the reduced maintenance for the inlet and column, we recommend determination of these analytes by headspace methods.

## Ethics statements

The authors have read and followed the ethical requirements for publication in *MethodsX*. Neither human study participants, animals, nor social media platforms were involved in collecting the data presented here.

## CRediT authorship contribution statement

**Janel E. Owens:** Methodology, Data curation, Validation, Writing – review & editing, Funding acquisition. **Luis E. Lowe:** Conceptualization, Data curation, Formal analysis, Writing – original draft, Visualization.

## Declaration of Competing Interest

The authors declare that they have no known competing financial interests or personal relationships that could have appeared to influence the work reported in this paper.

## Data Availability

Data will be made available on request.
